# Development and validation of the Vietnamese Primary Care Assessment Tool – provider version

**DOI:** 10.1017/S1463423619000458

**Published:** 2019-07-01

**Authors:** Nguyen Thi Hoa, Anselme Derese, Jeffrey F. Markuns, Nguyen Minha Tam, Wim Peersman

**Affiliations:** 1 Department of Family Medicine, Hue University of Medicine and Pharmacy, Hue University, Hue, Vietnam; 2 Department of Public Health and Primary Care, Campus UZ 6K3, Gent, Belgium; 3 Global Health Collaborative, Department of Family Medicine, Boston University, Boston, MA, USA; 4 Research Group Social Work, Odisee University College, Brussel, Belgium; 5 Department of Rehabilitation Sciences, Ghent University, Campus UZ, Gent, Belgium

**Keywords:** assessment, primary care quality, provider perspectives

## Abstract

**Aim:**

To adapt the provider version of the Primary Care Assessment Tool (PCAT) for Vietnam and determine its internal consistency and validity.

**Background:**

There is a growing need to measure and explore the impact of various characteristics of health care systems on the quality of primary care. It would provide the best evidence for policy makers if these evaluations come from both the demand and supply sides of the health care sector. Comparatively more researchers have studied primary care quality from the consumer perspective than from the provider’s perspective. This study aims at the latter.

**Method:**

Our study translated and adapted the PCAT provider version (PCAT PE) into a Vietnamese version, after which a cross-sectional survey was conducted to examine the feasibility, internal consistency and validity of the Vietnamese PCAT provider version (VN PCAT PE). All general doctors working at 152 commune health centres in Thua Thien Hue province had been selected to participate in the survey.

**Findings:**

The VN PCAT PE is an instrument for evaluation of primary care in Vietnam with 116 items comprising six scales representing four core primary care domains, and three additional scales representing three derivative domains. From the translation and cultural adaptation stage, two items were combined, two items were removed and one item was added. Six other items were excluded due to problems in item-total correlations. All items have a low non-response or ‘don’t know/don’t remember’ response rate, and there were no floor or ceiling effects. All scales had a Cronbach’s alpha above 0.80, except for the Coordination scale, which still was above the minimum level of 0.70.

**Conclusion:**

The VN PCAT PE demonstrates adequate internal consistency and validity to be used as an effective tool for measuring the quality of primary care in Vietnam from the provider perspective.

## Introduction

Since the Alma-Ata declaration 40 years ago, primary care has been described repeatedly as essential care that is (1) universally accessible to individuals and families in communities, (2) available at an affordable cost to communities and countries and (3) the first level of contact for patients (or the first element of a continuing health care process) (WHO, 2008). With these notable features, there is compelling evidence that stronger primary care systems are associated in general with better population health outcomes including lower mortality rates, rates of premature death and hospitalizations for ambulatory care sensitive conditions, and higher infant birth weight, life expectancy, and satisfaction with the health care system (Starfield, 1991; Starfield and Shi, 2002; Macinko *et al.*, 2003; Niti and Ng, 2003). Primary care is a factor in improving public health and health outcomes and the prevention of illness and death, with lower use of hospital-based medical care, associated with lower costs (Starfield *et al.*, 2005b; Friedberg *et al.*, 2010), and more equitable distribution of health within a population (Starfield *et al.*, 2005a; 2005b; Shi *et al.*, 2005a; 2005b). A critical review on the contribution of primary care to health and health systems in low- and middle-income countries (LMIC) showed that primary-care-focused health initiatives have improved access to health care, including among the poor, at reasonably low cost (Kruk *et al.*, 2010). There is also evidence that primary care programmes have reduced child mortality and, in some cases, wealth-based disparities in mortality (Kruk *et al.*, 2010).

Similar to many LMIC, Vietnam faces the challenges of the double burden of communicable and non-communicable disease and the trend to sustainable development from its own funding. Since 2013, the government has issued many important policy changes to reinforce the grassroot networks as well as the health care system in general (Vietnam Ministry of Health, 2013; Vietnam Prime Minister, 2013; Prime Minister, 2016; Vietnam Government, 2016; Ministry of Health, 2016a; 2016b; 2017).

In 2015, the Primary Health Care Performance Initiative (PHCPI) was launched in 135 LMIC with the aim of catalyzing improvements in primary health care systems (PHCPI). The PHCPI conceptual framework conceived of a high-quality primary health care subdomain, which includes the classic primary health care functions such as first contact accessibility, comprehensiveness, and coordination as first laid out by Starfield and others in the world plus added a new function in person-centred care to distinguish between the continuity and person-centred components in Starfield’s original domain of person-focused care over time. This high-quality primary care is one of the key subdomains for measurement of primary health care service delivery in health systems (Veillard *et al.*, 2017).

Worldwide, commitment for improvements in primary care is increasing. An example is the new UN Sustainable Goal for Health (Enhance health and promote well-being for all at all ages) (World Health Oganization, 2016). Recently, the new Astana Declaration: ‘From Alma-Ata towards universal health coverage and the Sustainable Development Goals’ released by WHO and UNICEF in October 2018 reaffirmed the commitment of States and Governments to ‘build a sustainable primary health care as well as to enhance capacity and infrastructure for primary care – the first contact with health services’ (WHO and UNICEF, 2018). Consequently, there is also a growing need to measure various characteristics of primary care as we mentioned above and explore their impact on the quality of primary care. It would provide the best evidence for policy makers if these evaluations come from both the demand and supply sides of the health care sector. Comparatively more researchers have studied assessments of primary care quality from the consumer perspective than from the workforce perspective. A recent South African study pointed out that there is a significant gap between the two, that is, between the clients’ experience with primary care and what managers and providers think they are delivering (Bresick *et al.*, 2016).

There are various tools that have been used for measuring characteristics of primary care, for example, the CPCI (Components of Primary Care Instrument) (Flocke, 1997), the PCAS (Primary Care Assessment Survey) (Safran *et al.*, 1998), the EUROPEP questionnaire (European Task Force on Patient Evaluations of General Practice Care) (Grol *et al.*, 1999), the CAHPS (Consumer Assessment of Healthcare Providers and Systems) (Weidmer *et al.*, 2014), the P3 C (Parents’ Perception of Primary Care) (Seid *et al.*, 2001), and the PCAT (Primary Care Assessment Tool) (Shi *et al.*, 2001). The PCAT developed by Barbara Starfield at the Johns Hopkins Primary Care Policy Centre is one of the most widely studied and applied tools for measuring the quality of primary care across the globe. The PCAT family includes four versions: the consumer–client, facility, provider and health system versions. Through the PCAT, primary care quality is evaluated according to its core principles (first contact care, continuous longitudinal care, coordination, and comprehensiveness) and three other derivative domains (family-centered care, community-orientated care, and culturally competent care) (Malouin *et al.*, 2009). In contrast with the consumer version, which has been translated and validated in many languages and countries across the world (Rocha *et al.*, 2012; Yang *et al.*, 2013; Wang and Shi, 2014; Aoki *et al.*, 2016), little work has been done for the provider version questionnaires.

As the PCAT consumer version was validated and successfully used in Vietnam (Hoa *et al.*, 2018), we found that the PCAT provider version could render an adequate reflection on organizational resources and health care processes from a primary care provider perspective. As a first step, this study was conducted to adapt the PCAT provider tool for Vietnam and determine its internal consistency and validity.

## Method

### Translation and adaptation of the PCAT provider version for Vietnam

**Figure 1.**
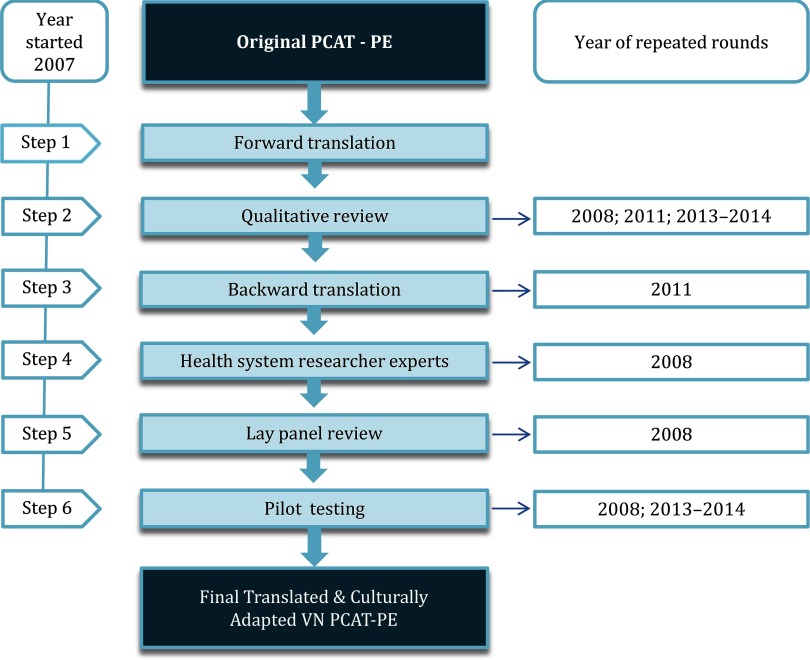
Process of translation and cultural adaptation for VN PCAT-PE

The PCAT provider version (PCAT PE) was translated and culturally adapted strictly according to the guidelines from the Johns Hopkins Primary Care Policy Center for use in international settings (Starfield and Shi, 2009) (illustrated by Figure 1). The first round was done in 2007 including all recommended steps as follows:

- **Step 1:** *Forward translation* performed by a bilingual physician and PhD student whose native tongue was Vietnamese with experience in translating documents between English and Vietnamese. This translator was familiar with use of the PCAT. To the best of the translator’s ability, the translation preserved the intent rather than the literal meaning of the items.
- **Step 2:** *Qualitative review* of the translated survey was done by several doctors and other workers from Hanoi Medical School. This was performed in focus group discussion, where every translated item was reviewed to ensure its clarity, use of common language, and conceptual adequacy.
- **Step 3:** *Backward translation* was done by a Vietnamese woman whose native language is American English and who has lived long enough in the USA to know the language and routines of daily life. This translator was not familiar with the specific wording of the original PCAT terms. The instructions given to the back translator were identical to those given to the forward translator. The aim of this step was to identify items that required further study.
- **Step 4:** *Health systems research experts and the forward/backward translators jointly reviewed* the forward and backward translations in order to detect items that were not effectively translated, which were confusing or generated concerns. A few modifications were made until a consensus version was reached.
- **Step 5:** Thereafter a *lay panel of Vietnamese physicians reviewed* the translation, identified troublesome items, and proposed alternatives.
- **Step 6:** *Pilot testing* of the translated version: the questionnaire was administered to 108 physicians, that is, 41 physicians working at Commune health centers (CHCs) and 67 physicians working as academic trainers and administrators at the medical universities. Basic descriptive analyses were conducted to ensure adequate distribution of responses. The respondents were debriefed to identify any wording or comprehension problems.

To ensure the high quality of the questionnaires, certain steps were repeated in 2008 (steps 6, 2, 4, 5), 2011 (steps 2 and 3), 2013, and 2014 (steps 2 and 6) before it was declared fit to be used in a general population (Table 1). Below we describe those steps with the year wherein they were performed:

**Table 1.** Different steps in the translation and adaptation process and in which rounds they were repeated

Step	Round 1, 2007	Round 2, 2008	Round 3, 2011	Round 4, 2013 and 2014
Step 1: Forward translation	x			
Step 2: Qualitative review	x	x	x	x
Step 3: Backward translation	x		x	
Step 4: Health system researcher experts	x	x with Dr. Barbara Starfield’s comments		
Step 5: Lay panel review	x	x		
Step 6: Pilot testing	x	x		x

In 2008, *Pilot testing* was performed again for 28 physicians in the Specialist Level 1 in family medicine (CK1) training programme in Khanh Hoa. A dissemination workshop was then held in Vietnam with primary care physicians from several medical schools to review the pilot data and make additional revision suggestions based on responses from the previous pilot testing round (*Qualitative review*).

Following this review, a panel of primary care physicians from six medical schools in Vietnam and a team of researchers and physicians from Boston University participated in two rounds of revisions of PCAT questions, including appropriate contextual translation of concepts (*Lay panel review*).

Dr. Barbara Starfield reviewed the revised version pre-translation and gave comments that were incorporated into a final version (*Health system researcher experts review*).

In 2011, a *Qualitative review* was repeated by the research team (Hue UMP and BU). Discussion on the cultural relevance of each item in the Vietnamese version and comparison between the current version and the original PCAT were made. This round also checked the matching between each equivalent item of the consumer and provider surveys. The research team produced a list of problematic items and proposed solutions. *Backward translation* was repeated after the qualitative review. The back translation was undertaken by a woman whose native language is American English and has lived in the USA long enough to know the language and routines of daily life. A new translated version of the questionnaires was produced.

In April 2013, *an additional pilot study* was conducted for 60 physicians working at CHCs in Thua Thien Hue Province. These physicians were divided into two groups: one group read the questionnaires and gave their opinions in terms of content and accuracy of evaluation for practice of physicians working in primary care in Vietnam. The other group was asked to fill in the entire questionnaire and give their feedback on challenges they faced.

From October 2013 to January 2014, a final revision was done by the research team from Hue UMP and BU (*qualitative review*). The team went through all the items and asked for advice from international experts with experience in PCAT validation. After this round, *a final translated version of the questionnaire* was produced with 9 scales and 123 items as compared to 9 scales and 124 items of the original PCAT provider. This is a self-completion questionnaire and takes approximately 30–45 min to fulfil. We maintained a four-point Likert scale response format (1 = definitely not; 2 = probably not; 3 = probably; and 4 = definitely) providing an additional ‘don’t know/don’t remember’ option in case participants could not choose one of those four options. Table 1 in Supplementary Material shows items changed in the final translated questionnaires from the original version.

### Data collection

To evaluate the feasibility, internal consistency, and validity of the VN PCAT PE, a cross-sectional study was implemented. The study was conducted in Thua Thien Hue province with all general doctors working at CHCs. There are 152 CHCs in the 9 districts of this province. Normally, one CHC is equipped with a general doctor as the head of the CHC. There are some exceptions: some CHCs have two general doctors, others only a traditional medicine doctor or an assistant traditional medicine doctor or an assistant doctor.

The questionnaires were delivered at the end of the monthly meeting of each district health center. In cases where one or more doctors were absent in that meeting, we tried to contact them and make an appointment at their CHC to have an interview at a later stage, where a trained interviewer assisted the doctor to complete the questionnaire. After three unsuccessful engagement efforts during the study period, we excluded these doctors from our research. Before the interview, participants received a full explanation of the study’s content and purpose and signed a consent form if they agreed to participate. Participants received 5 USD as an appreciation gift for their time and contribution. Data collection was conducted from December 2017 to February 2018.

This study obtained ethical approval from the Scientific Committee of Hue University of Medicine and Pharmacy on 18 March 2014 and IRB review from Boston University (H-31432).

### Data analysis

All collected questionnaires were cleaned and entered into EpiData. Data analysis was performed using SPSS software version 23.0.

**Figure 2.**
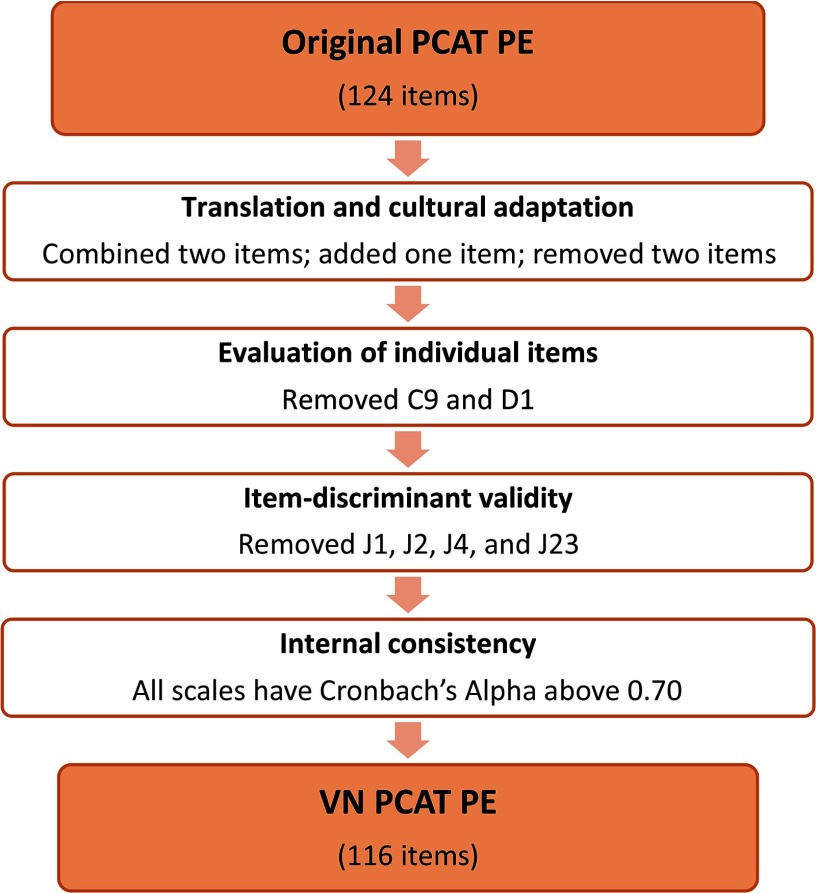
Validation process of VN PCAT-PE and its results

Subsequent full validation involved several steps (Figure 2). First, individual items were evaluated on several criteria. Items with a high percentage (≥20%) of item non-response or ‘don’t know/don’t remember’ responses, or items with a large floor or ceiling effect (>80% of respondents chose the lowest or highest rating) were removed. Next, the item-total correlation for the remaining items in each scale was calculated (item-total correlation before review). Items were removed if the item-total correlation was below 0.30 or if Cronbach’s coefficient alpha for that scale improved substantially when the item was removed. Finally, item-discriminant validity was tested: for each item, the item-total correlation (item-total correlation after review) with the hypothesized scale should be substantially higher than the correlation with the other scales. In the second phase, Cronbach’s coefficient alpha was used to examine how well all items measured the same construct (internal consistency). A value of 0.70 is commonly seen as a minimum.

The recoding progress and calculation for the sum mean score of domains and subdomains of primary care strictly complied with the guideline PCAT manual issued by Johns Hopkins University in 1998. For calculating the sum mean scores of domains and subdomains, a mean value was assigned to ‘not sure/don’t remember’ answers as well as to missing values.

## Results

### Characteristics of study population

Among the 157 doctors working at the 152 CHCs in Thua Thien Hue province, 150 participated in our study, one refused and six were absent because of maternal or sick leave or study leave. Tables 2 and 3 show the characteristics of the participants and their work place. There were about twice as many male doctors as female ones. More than half of these doctors have been practicing for 20 years or more. Although CHCs receive patients of all ages, the majority of them are adults and only a small percentage of them must pay out-of-pocket for their health visits.

**Table 2.** Characteristics of study population-providers (*n* = 150)

Characteristics	*n*	%
Gender		
Female	52	34.7
Male	98	65.3
Age Mean 46.2, SD 7.85, Min 29, Max 60		
29–39 year old	33	22.0
40–50 year old	62	41.3
51–60 year old	55	36.7
Number of years in practice Mean 18.32, SD 9.3, Min 1, Max 35		
<10 years	35	23.3
10–19 years	24	16.0
20–29 years	83	55.3
30 years and more	8	5.3

**Table 3.** Characteristics of study population-health facilities (*n* = 150)

Characteristics	Mean (SD)	Min	Max
Number of consultations per day	28.72 (14.2)	5	95
Number of consultations per week	155.67 (86.5)	10	500
Percentage of consultations by age			
0–6 year old	19.95 (14.2)	0	100
7–16 year old	15.21 (8.5)	0	50
17–59 year old	34.89 (18.2)	0	85
60–80 year old	20.52 (11.2)	0	60
>80 year old	10.12 (8.1)	0	50
Percentage of patients with chronic problems (mental and physical)	***N***	%	
<20%	86	62.8	
From 20% to 40%	37	27.0	
From 41% to 60%	10	7.3	
>60%	4	2.9	
Payment resources of patients	Mean (SD)	Min	Max
Government health insurance	93.7 (12.3)	40	100
Out of pocket	3.7 (8.9)	0	50

### Evaluation of the individual items

Table 4 shows the evaluation of the individual items. All items have a low non-response or ‘don’t know/ don’t remember’ response rate (<20%) and there were no floor or ceiling effects (≤80%). One item from First contact access (C9) and one item from Ongoing care (D1) were removed because of an item-total correlation below 0.30. The Cronbach’s alphas of the different scales were not improved substantially by removing any items. Four items of the Community orientation care scale were removed because their item-total correlation with that scale was lower than their correlations with the other scales. (see Table 2 - Supplementary Material).

**Table 4.** Item mean (SD), percentage ‘don’t know, don’t remember/missing,’ floor/ceiling effect, item-total correlation before review, item-total correlation after review, and range of item correlations with other domains

Item code in the data set	Item	Item mean (SD)	% Missing/ % don’t know, don’t remember	% Floor/ceiling effect	Item-total correlation before review	Item-total correlation after review	Range of item correlation with other domains
	C. First contact – Access (nine items)						
C1	Is your office open on Saturday or Sunday?	3.15 (1.34)	0.0/2.0	14.7/41.3	0.73	0.75	0.08/0.26
C2	Is your office open on at least some weekday evenings until 8pm?	3.05 (1.49)	0.0/2.7	17.3/38.0	0.71	0.73	−0.04/0.14
C3	When your office is open, and patients get sick, would someone from your office see them that day?	3.54 (0.61)	0.0/0.0	0.7/59.3	0.45	0.45	0.07/0.27
C4	When your office is open, can patients get advice quickly over the phone when they think they need it?	2.98 (0.89)	0.0/0.0	4.7/32.7	0.62	0.62	0.16/0.30
C5	When your office is closed, can patients contact you or another doctor by phone when they get sick?	3.07 (0.98)	0.0/0.7	1.3/35.3	0.70	0.70	0.14/0.29
C6	If your office is closed on Saturday or Sunday and patients get sick, would someone from your office be able to see them that day?	3.03 (1.19)	0.7/1.3	9.3/34.0	0.75	0.75	0.13/0.24
C7	When your office is closed during the night and patients get sick, would someone from your office be able to see them that night?	3.14 (1.30)	0.7/2.0	11.3/38.0	0.66	0.66	0.07/0.23
C8	Can a patient easily get an appointment or make a visit for routine check-ups at your office?	3.31 (0.86)	0.0/0.7	0.7/42.0	0.55	0.56	0.20/0.35
C9	On average, do patients have to wait more than 30 min after arriving before they are examined by the doctor or nurse?	1.74 (0.96)	1.3/0.7	43.3/4.0	0.22	Not assessed	Not assessed
	D. Ongoing care (13 items)						
D1	At your office, do patients see the same clinician each time they make a visit?	3.10 (0.70)	0.0/0.0	2.7/27.3	0.23	Not assessed	Not assessed
D2	Can you understand the questions that your patients ask you?	3.66 (0.57)	2.7/0.0	0.7/68.7	0.50	0.49	0.15/0.25
D3	Do you think your patients understand what you ask them or say to them?	3.49 (0.78)	0.0/0.7	0.7/52.0	0.55	0.57	0.08/0.30
D4	If patients have a question, can they call and talk to the doctor or nurse who knows them best?	3.29 (0.90)	0.7/0.7	0.7/43.3	0.53	0.52	0.18/0.36
D5	Do you think you give patients enough time to talk about their worries or problems?	3.24 (0.86)	0.0/0.7	0.0/37.3	0.54	0.54	0.21/0.35
D6	Do you think your patients feel comfortable telling you about their worries or problems?	3.33 (0.81)	0.0/0.7	0.0/40.0	0.53	0.53	0.17/0.44
D7	Do you think you know the patients in your practice ‘very well’ (for example, both health condition and personal life)?	2.99 (1.22)	0.0/2.7	0.0/18.0	0.60	0.59	0.02/0.41
D8	Do you know who lives with each of your patients?	2.96 (1.16)	0.0/2.0	2.0/20.7	0.60	0.61	0.04/0.33
D9	Do you think you understand what problems are most important to the patients you see?	3.42 (1.56)	0.7/6.0	0.0/23.3	0.66	0.66	0.09/0.47
D10	Do you think you know each patient’s complete medical history?	2.97 (0.88)	0.0/0.7	1.3/22.0	0.70	0.70	0.05/0.38
D11	Do you think you know each patient’s work or employment?	3.08 (1.13)	0.0/2.0	2.0/23.3	0.67	0.69	0.07/0.47
D12	Would you know if patients had trouble getting or paying for a prescribed medication?	3.05 (1.52)	0.7/4.0	7.3/25.3	0.66	0.66	0.20/0.48
D13	Do you know all the medications that your patients are taking?	3.13 (1.14)	0.0/2.0	2.0/28.7	0.61	0.61	0.16/0.37
	E. Coordination (seven items)						
E2	Does your office share the results of the tests with patients (by phone call, mail, computer, or in person)?	2.90 (1.30)	0.7/2.7	5.3/17.3	0.45	0.45	0.02/0.33
E3	Do you think you know about all the visits that your patients make to specialists or special services?	2.69 (1.58)	0.0/4.7	6.0/9.3	0.61	0.61	0.26/0.45
E4	When patients need to be referred to a specialist, do you discuss with them the options available to get help for their problem?	3.12 (0.87)	0.0/0.7	1.3/29.3	0.69	0.69	0.22/0.52
E5	Does someone at your office help the patient make the appointment for the referral visit?	3.06 (1.79)	0.7/6.7	7.3/14.0	0.54	0.54	0.11/0.37
E6	When patients are referred, do you give them any written information to take to the specialist?	2.59 (1.50)	1.3/3.3	16.0/12.7	0.75	0.75	0.20/0.38
E7	Do you receive useful information about your referred patients back from the specialists or special services?	2.16 (1.59)	0.0/4.0	32.0/3.3	0.63	0.63	0.12/0.30
E8	Do you talk with your patients about their visit to specialists and the results of the visits to the specialist or special service?	2.81 (1.33)	0.7/2.7	8.0/16.0	0.66	0.66	0.10/0.43
	F. Coordination (information system) (nine items)						
F1	Do all patients have a medical record at the facility?	2.55 (1.76)	1.3/4.7	24.7/16.7	0.60	0.60	0.06/0.16
F2	Do patients have a medical record or booklet that they keep with them and bring to visits?	3.14 (1.36)	1.3/3.3	4.0/24.7	0.47	0.47	0.07/0.26
F3	Would you allow patients to look at their medical records at your office if they wanted to?	2.19 (1.58)	1.3/3.3	36.0/8.7	0.35	0.35	0.03/0.32
F4	Are patient records available when you see patients? (either personal or facility records)	3.02 (1.07)	1.3/0.7	10.0/32.7	0.67	0.67	0.10/0.29
	*Do you use the following methods to assure that indicated services are provided?*						
F5	Flow sheets in patients’ charts for lab results	2.63 (1.59)	0.0/4.0	20.0/10.0	0.65	0.65	0.10/0.40
F6	Printed guidelines in patients’ records	2.44 (1.63)	0.7/4.0	26.7/8.7	0.78	0.78	0.18/0.39
F7	Periodic medical record audits	2.59 (1.63)	0.0/4.0	24.7/12.0	0.83	0.82	0.25/0.46
F8	Problem lists in patients’ records	2.54 (1.66)	0.0/4.0	26.7/13.3	0.82	0.82	0.15/0.44
F9	List of medications patients are taking	2.80 (1.41)	0.0/2.7	14.7/20.7	0.78	0.78	0.20/0.41
	G. Comprehensiveness (services available)24 items						
	If patients need any of the following services, would they be able to get them on-site at your office?						
G1	Nutrition counselling	3.19 (0.74)	0.0/0.0	0.0/38.0	0.57	0.57	0.32/0.44
G2	Immunizations	3.52 (0.56)	0.7/0.0	0.0/54.7	0.56	0.56	0.17/0.42
G3	Assistance with obtaining available social service programmes/benefits	2.59 (1.40)	0.0/2.7	13.3/16.0	0.49	0.49	0.21/0.37
G4	Dental check-ups and dental treatments	2.55 (1.01)	0.0/0.7	8.0/16.0	0.52	0.52	0.15/0.40
G5	Family planning or birth control services	3.42 (0.78)	0.7/0.7	0.7/44.7	0.64	0.64	0.25/0.48
G6	Substance or drug abuse counselling or treatment	3.27 (0.62)	0.0/0.0	0.0/36.7	0.68	0.68	0.20/0.50
G7	Counselling for behaviour or mental health problems	3.13 (0.85)	0.0/0.7	0.0/28.7	0.72	0.72	0.29/0.51
G8	Counselling and treating alcoholism	2.78 (1.00)	0.0/0.7	4.7/22.0	0.68	0.68	0.15/0.51
G9	Suturing for a minor laceration	3.27 (0.88)	0.0/0.7	1.3/40.0	0.65	0.65	0.18/0.40
G10	Counselling and testing for HIV/AIDS	3.23 (1.16)	0.0/2.0	4.7/34.0	0.63	0.63	0.22/0.40
G11	Tympanocentesis	2.09 (1.79)	0.0/4.7	48.0/8.7	0.59	0.59	0.09/0.40
G12	Vision screening	2.89 (0.95)	0.7/0.7	2.0/23.3	0.69	0.68	0.24/0.46
G13	Allergy shots	3.21 (0.67)	0.0/0.0	0.0/35.3	0.68	0.68	0.18/0.48
G14	Temporary fix for broken bone	2.97 (0.89)	0.0/0.0	4.7/33.3	0.72	0.72	0.28/0.43
G15	Gastric catheter insertion	1.89 (1.59)	0.7/3.3	54.0/7.3	0.49	0.49	0.13/0.39
G16	Pap smears, cervical cancer screening	2.15 (2.16)	0.0/8.0	54.7/3.3	0.35	0.35	0.06/0.29
G17	Rectal exam or colon cancer screening	2.48 (2.87)	0.7/15.3	69.3/3.3	0.37	0.37	0.04/0.34
G18	Smoking counselling	2.95 (0.81)	1.3/0.0	2.7/26.7	0.70	0.70	0.12/0.55
G19	Prenatal care	3.15 (0.92)	0.0/0.7	2.0/34.7	0.73	0.73	0.22/0.46
G20	Shoulder reduction	1.95 (1.41)	0.7/2.0	46.7/11.3	0.65	0.65	0.11/0.38
G21	Advice on end of life issues/palliative care	2.22 (1.78)	0.0/4.7	40.0/10.0	0.57	0.56	0.06/0.42
G22	Advice on preparing for changes consequent to aging	2.50 (1.15)	0.0/1.3	10.7/14.7	0.62	0.62	0.18/0.57
G23	Postpartum care of umbilical cord	3.02 (0.78)	0.0/0.0	2.0/29.3	0.70	0.70	0.25/0.46
G24	Monitoring of normal pregnancy	3.46 (0.79)	2.7/0.7	0.7/48.0	0.64	0.64	0.21/0.36
	H. Comprehensiveness (services provided) 18 items						
	If your office serves all ages, please answer all questions in this section (H1–H18). If your office serves only children, do not answer questions H3–H12.If your office serves only adults, do not answer questions H12–H17						
	Are the following subjects discussed with patients?						
H1	Nutritional/non-nutritional foods or getting enough sleep	2.85 (0.93)	0.7/0.7	2.0/20.7	0.64	0.64	0.19/0.51
H2	Home safety, such as storing medicines safely	3.03 (1.15)	0.7/2.0	5.3/19.3	0.51	0.51	0.18/0.37
	Questions H3–H12 apply to adults only (ages 18 and older)						
	Are the following subjects discussed with patients?						
H3	Seat belt or helmet use	2.42 (1.25)	2.7/1.3	21.3/14.7	0.63	0.63	0.12/0.48
H4	Handling family conflicts	2.28 (1.49)	0.7/3.3	22.0/7.3	0.55	0.55	0.09/0.51
H5	Advice about appropriate exercise	2.79 (1.15)	0.0/2.0	3.3/12.0	0.63	0.63	0.11/0.50
H6	Cholesterol levels	2.48 (1.39)	0.7/2.7	16.0/10.7	0.55	0.54	0.11/0.39
H7	Medications being taken	3.05 (0.68)	0.0/0.0	0.7/25.3	0.61	0.61	0.13/0.49
H8	Exposure to harmful substances at home, work, or in their neighbourhood	2.58 (0.96)	0.0/0.7	3.3/16.7	0.74	0.74	0.14/0.58
	Gun availability, storage, safety	Removed	Removed	Removed			
H9	Prevention of hot water burns	2.89 (1.05)	0.0/1.3	0.7/21.3	0.81	0.81	0.16/0.61
H10	Prevention of falls	2.78 (0.77)	0.0/0.0	0.7/20.0	0.80	0.80	0.07/0.63
H11	Prevention of osteoporosis or fragile bones in females	2.76 (0.93)	0.0/0.7	3.3/16.7	0.70	0.70	0.09/0.52
H12	Care for common menstrual or menopausal problems	2.69 (1.07)	0.0/1.3	3.3/15.3	0.73	0.73	0.13/0.55
	Questions H13–H17 apply to children only (under age 18) Are the following subjects discussed with the child and parent/guardian?						
H13	Ways to handle problems with child’s behaviour	2.39 (0.75)	0.0/0.0	6.7/9.3	0.77	0.77	0.22/0.64
H14	Changes in growth and behaviour that parents can expect at certain ages	2.51 (0.71)	0.0/0.0	4.0/8.7	0.71	0.71	0.17/0.63
H15	Safety issues for children under 6: (injury prevention, fire and electricity safety, food safety, drowning prevention)	2.70 (0.74)	0.0/0.0	2.0/14.7	0.76	0.76	0.18/0.66
H16	Safety issues for children between 6 and 12: (including using helmets and/or seatbelts)	2.33 (0.87)	0.0/0.0	15.3/11.3	0.74	0.74	0.11/0.66
H17	Safety issues for children over 12: safe sex, saying no to drugs, not drinking and driving	2.41 (0.99)	0.0/0.7	10.7/12.0	0.70	0.70	0.10/0.66
	I. Family centeredness (14 items)						
I1	Does your office ask patients about their ideas and opinions when planning treatment and care for the patient or family member?	2.72 (0.73)	0.0/0.0	4.0/12.7	0.64	0.64	0.17/0.43
I2	Does your office ask about illnesses or problems that might run in the patients’ families?	2.85(0.70)	0.0/0.0	0.7/17.3	0.71	0.71	0.15/0.59
I3	Is your office willing and able to meet with family members to discuss a health or family problem?	3.08 (0.67)	0.0/0.0	0.7/26.0	0.61	0.61	0.15/0.47
	Are the following included as a routine part of your health assessment?						
	Use of familiograms, family APGAR	Removed	Removed	Removed			
I4	Discussion of family health risk factors, for example., genetics	2.67 (0.94)	0.0/0.7	3.3/15.3	0.67	0.67	0.13/0.56
I5	Discussion of family economic resources	2.35 (1.06)	0.0/1.3	8.7/8.0	0.78	0.78	0.09/0.63
I6	Discussion of social risk factors, for example, loss of employment	2.23 (1.20)	0.0/2.0	15.3/4.7	0.75	0.75	0.05/0.55
I7	Discussion of living conditions (eg, clean water, latrine/toilet, stress at work or home)	2.82 (0.72)	0.0/0.0	0.7/18.0	0.76	0.76	0.16/0.63
I8	Discussion of health status of other family members	2.58 (0.86)	0.7/0.7	2.0/8.7	0.77	0.77	0.14/0.60
I9	Discussion of parenting	2.40 (0.90)	0.7/0.7	5.3/8.7	0.78	0.78	0.05/0.68
I10	Assessment of signs of child abuse	2.24 (1.24)	0.0/2.0	17.3/7.3	0.78	0.77	0.01/0.58
I11	Assessment of indications of family in crisis	2.17 (1.33)	0.0/2.7	20.7/4.7	0.75	0.75	0.02/0.57
I12	Assessment of impact of patient’s health on family functioning	2.41 (1.21)	0.0/2.0	10.7/8.0	0.78	0.78	0.10/0.67
I13	Assessment of development level	2.85 (0.90)	0.0/0.7	3.3/16.7	0.63	0.63	0.29/0.55
	J. Community orientation (21 items)						
J1	Does your office make home visits?	2.27 (0.62)	0.0/0.0	2.0/7.3	0.44	0.44	0.00/0.48
J2	Do you think your office has adequate knowledge about the health problems of the communities you serve?	2.92 (1.42)	0.0/4.0	2.7/10.0	0.41	0.41	0.14/0.48
J3	Does your office get opinions and ideas from people that might help to provide better health care?	2.79 (0.90)	1.3/0.7	2.0/15.3	0.59	0.59	0.19/0.45
J4	Is your office able to change health care services or programmes in response to specific health problems in the communities?	2.70 (1.31)	0.0/2.7	8.0/11.3	0.45	0.45	0.10/0.45
	Does your office use the following types of data to determine what programmes/services are needed by the communities you serve?						
J5	Mortality data (data on deaths)	3.33 (0.82)	0.0/0.7	0.7/40.7	0.47	0.47	0.12/0.42
J6	Public health communicable disease data (eg, STDs, TB)	3.27 (0.62)	0.0/0.0	0.7/35.3	0.53	0.53	0.20/0.43
J7	Community immunization rates	3.59 (0.68)	0.0/0.7	0.0/55.3	0.51	0.51	0.25/0.44
J8	Public health data on health or occupational hazards	3.03 (0.75)	0.0/0.0	1.3/28.0	0.59	0.59	0.08/0.48
J9	Clinical data from your practice	3.14 (1.02)	0.0/1.3	3.3/28.0	0.6	0.60	0.16/0.42
	Does your office use the following methods to monitor and/or evaluate the effectiveness of services/programmes?						
J11	Surveys of your patients	2.63 (0.82)	0.0/0.0	5.3/16.7	0.73	0.73	0.14/0.41
J12	Community surveys	2.59 (0.80)	0.7/0.0	4.7/14.7	0.74	0.74	0.06/0.39
J13	Feedback from community organizations or community advisory boards	2.51 (0.95)	0.0/0.7	6.7/11.3	0.79	0.79	0.08/0.52
J14	Feedback from your practice staff	2.72 (0.76)	0.7/0.0	4.0/14.7	0.73	0.73	0.20/0.50
J15	Analysis of local data or vital statistics	2.95 (1.18)	0.0/2.0	4.7/20.0	0.76	0.76	0.05/0.51
J16	Systematic evaluations of your programmes and services provided	2.85 (0.95)	0.0/0.7	4.7/20.0	0.77	0.77	0.13/0.56
J17	Community/village health workers	3.05 (0.77)	0.0/0.0	4.0/28.0	0.63	0.63	0.16/0.40
J18	Gather feedback from patients about health staff performance	2.78 (0.79)	0.0/0.0	4.7/18.0	0.67	0.67	0.07/0.43
	Does your office use any of the following activities to reach out to populations in the communities you serve?						
J20	Networking with state and local agencies involved with culturally diverse groups	2.75 (1.18)	0.0/1.3	12.7/19.3	0.64	0.64	0.18/0.47
J21	Linkages with religious organizations	2.29 (1.64)	0.0/4.0	30.7/8.7	0.67	0.67	0.12/0.41
J22	Involvement with neighbourhood groups/ community leaders	2.93 (1.51)	0.0/4.0	8.0/18.0	0.67	0.67	0.23/0.54
J23	Village health workers	3.26 (0.97)	0.7/1.3	2.7/32.0	0.38	0.38	0.15/0.42
	K. Culturally competent (nine items)						
K1	Can someone in your office communicate well with patients who speak another language (such as patients from ethnic minority groups)?	2.74 (2.19)	0.0/8.0	40.7/20.0	0.58	0.58	0.07/0.34
K2	Do you take into account a family’s special beliefs about health care or use of folk medicine, such as herbs/homemade medicines?	2.99 (1.15)	0.0/2.0	4.7/18.0	0.55	0.55	0.22/0.39
K3	Do you take into account a family’s request to use alternative treatment, such as homeopathy or acupuncture?	2.97 (0.83)	0.0/0.7	1.3/17.3	0.49	0.49	0.23/0.47
	Does your office use any of the following methods to address the cultural diversity in your patient population?						
K4	Training of staff by outside instructors	2.27 (1.60)	0.0/4.0	27.3/6.0	0.68	0.68	0.15/0.48
K5	In-service programmes presented by staff	2.59 (1.65)	0.0/4.7	18.0/7.3	0.64	0.64	0.08/0.41
K6	Use of culturally sensitive (language, visual images, religious customs) materials/pamphlets	2.65 (1.11)	0.0/1.3	10.0/12.0	0.73	0.73	0.13/0.48
K7	Staff reflecting the cultural diversity of the population served	2.57 (1.38)	0.7/2.7	16.7/9.3	0.73	0.73	0.20/0.43
K8	Translators/interpreters	2.15 (2.20)	0.7/8.0	58.0/5.3	0.65	0.65	0.08/0.29
K9	Planning of services that reflect cultural diversity	2.48 (2.00)	1.3/6.7	36.7/7.3	0.73	0.73	0.18/0.45

### Internal consistency of the different scales

Based on these parameters, 116 items of the VN PCAT-PE were determined to be appropriate for use with Vietnamese health care providers, to represent four core domains with six scales and three derivative domains with three scales (Table 5). All scales had a Cronbach’s alpha above 0.80, except for the scale of Coordination, which still was above the minimum level of 0.70.

**Table 5.** Descriptive statistics of the domains scales

Domains	Mean (SD)	Cronbach’s alpha	Number of items in the Vietnamese version (Total 116)	Number of items in the original version (Total 124)
First contact – Access	3.09 (0.60)	0.82	8	9
Ongoing Care	3.11 (0.44)	0.84	12	13
Coordination	2.53 (0.51)	0.73	7	7
Coordination (information system)	2.44 (0.64)	0.85	9	8
Comprehensiveness (services available)	2.70 (0.49)	0.93	24	25
Comprehensiveness (services provided)	2.58 (0.54)	0.93	17	18
Family Centeredness	2.50 (0.52)	0.93	13	14
Community Orientation	2.83 (0.51)	0.92	17	21
Culturally Competent	2.32 (0.57)	0.82	9	9

## Discussion

### Main findings

The outcome of this study is a translated and adapted PCAT provider version for Vietnam. The results showed that this questionnaire is a valid tool to evaluate primary care quality in Vietnam from the provider viewpoint with high overall reliability and validity.

### Interpretation of the results in relation to existing literature

This study rendered a PCAT ready for evaluation studies of the primary care system from the providers perspective in Vietnam. Previous PCAT validation studies focused mostly on the patients’ (consumers’) version. Now that the providers’ version is available, a deeper and more comprehensive assessment of primary care quality becomes possible, adding a second key view on the demand–supply relationship of the primary care system of Vietnam.

The VN PCAT provider version preserves the integrity characteristics of the original PCAT provider version with 116 items belonging to nine scales. There were only slight changes in the number of items in most scales except for the Community Orientation scale, from which four items were removed because their item-total correlation with the hypothesized scale was lower than the correlations with another scale.

In a South African study, a new scale (about the primary health care team) was added at the end of the questionnaire (Bresick *et al.*, 2016). A Chinese study removed the scale of First contact access from their tool (Zou *et al.*, 2015). We succeeded in retaining most major characteristics of the original tool, however, preserving the possibility of future comparison with other primary care quality assessment studies using the original PCAT tool.

In the validation study of the consumer tool VN PCAT AE, the domains of First contact access and Comprehensive (service available) more items were removed (six and five items, respectively) (Hoa *et al.*, 2018). A probable reason why this was not the case in the provider study is that the providers had more knowledge about the items’ content and knew better the services they were providing than the consumers. This may have reduced the ground effect and the number of ‘don’t know/ don’t remembers’ as well as the number of missing answers.

Due to the fact that Vietnam has a specific culture (mid-level country, Southeast-Asian) and a developing primary care context, the 2007 process alone was not sufficient. As the reader may have observed, it was indeed a lengthy process for the translation and cultural adaptation (from 2007 to 2014). In order to improve its quality, various important steps were repeated several times, including four times for the qualitative review and three times for pilot testing. These added steps were necessary to develop a well-constructed and fully adapted tool for measuring the specific health care setting of Vietnam.

There are several potential biases of this study due to its limitation in design: the study population was restricted to general doctors working at CHCs. Although they are the major resource for providing primary care in Vietnam currently, there are other primary care doctors such as private doctors and doctors working in primary care outpatient clinics of some hospitals who should also be surveyed to assure the expected diversity and comprehensiveness of the tool.

## Conclusions

We developed the VN PCAT PE as a valid and reliable tool to measure the quality of primary care from a provider perspective in Vietnam. Used together with the VN PCAT AE, primary care performance can be examined comprehensively. The gap in views between primary care users (demand side) and providers (supply side) in Vietnam can now be identified.

## Supplementary material

For supplementary material accompanying this paper visit https://doi.org/10.1017/S1463423619000458. click here to view supplementary material

## References

[ref1] Aoki T , Inoue M and Nakayama T (2016) Development and validation of the Japanese version of Primary Care Assessment Tool. Family Practice 33, 112–117. 26546033 10.1093/fampra/cmv087

[ref2] Bresick, GF , Sayed AR , Le Grange C , Bhagwan S , Manga N and Hellenberg D (2016) Western Cape Primary Care Assessment Tool (PCAT) study: measuring primary care organisation and performance in the Western Cape Province, South Africa (2013). African Journal of Primary Health Care & Family Medicine 8, e1–e12. 10.4102/phcfm.v8i1.1057 PMC4913443 27247157

[ref3] Flocke SA (1997) Measuring attributes of primary care: development of a new instrument. Journal of Family Practice 45, 64–74. 9228916

[ref4] Friedberg MW , Hussey PS and Schneider EC (2010) Primary care: a critical review of the evidence on quality and costs of health care. Health Affairs 29, 766–772. 20439859 10.1377/hlthaff.2010.0025

[ref5] Grol R , Wensing M , Mainz J , Ferreira P , Hearnshaw H , Hjortdahl P , Olesen F , Ribacke M , Spenser T and Szecsenyi J (1999) Patients’ priorities with respect to general practice care: an international comparison. European Task Force on Patient Evaluations of General Practice (EUROPEP). Family Practice 16, 4–11. 10321388 10.1093/fampra/16.1.4

[ref6] Hoa NT , Tam NM , Peersman W , Derese A and Markuns JF (2018) Development and validation of the Vietnamese primary care assessment tool. PloS One 13, e0191181. 29324851 10.1371/journal.pone.0191181 PMC5764365

[ref7] Kruk ME , Porignon D , Rockers PC and Van Lerberghe W (2010) The contribution of primary care to health and health systems in low- and middle-income countries: a critical review of major primary care initiatives. Social Science and Medicine 70, 904–911. 20089341 10.1016/j.socscimed.2009.11.025

[ref8] Macinko J , Starfield B and Shi L (2003) The contribution of primary care systems to health outcomes within Organization for Economic Cooperation and Development (OECD) countries, 1970–1998. Health Services Research 38, 831–865. 12822915 10.1111/1475-6773.00149 PMC1360919

[ref9] Malouin RA , Starfield B and Sepulveda MJ (2009) Evaluating the tools used to assess the medical home. Managed Care (Langhorne, Pa) 18, 44–48. 19569570

[ref10] Ministry of Health (2016a) Decision No. 1568/QD–BYT dated on April 27, 2016 on approval for the plan on expanding and develop ment of the family medicine clinic model in the period 2016–2020 (in Vietnamese). Phê duyệt kế hoạch nhân rộng và phát triển mô hình phòng khám Bác sĩ gia đình tại Việt Nam giai đoạn 2016–2020.

[ref11] Ministry of Health (2016b) Plan No. 139/KH-BYT dated on March 1, 2016 Plan for people’s health protection, care, and promotion in the period 2016–2020 (in Vietnamese). Ban hành kế hoạch bảo vệ, chăm sóc và nâng cao sức khoẻ nhân dân giai đoạn 2016–2020.

[ref12] Ministry of Health (2017) Circular No. 39/2017/TT-BYT dated on October 18, 2017 on Health on basic package of health services applied to grassroots health facilities (in Vietnamese). Thông tư quy định gói dịch vụ y tế cơ bản cho tuyến y tế cơ sở.

[ref13] Niti M and Ng TP (2003) Avoidable hospitalisation rates in Singapore, 1991–1998: assessing trends and inequities of quality in primary care. Journal of Epidemiology and Community Health 57, 17–22. 12490643 10.1136/jech.57.1.17 PMC1732279

[ref14] Primary Health Care Performance Initiative. Primary Health Care Performance Initiative. Retrieved from http://www.phcperformanceinitiative.org/ [accessed 6 October 2018].

[ref15] Prime Minister (2016) Decision No. 2348/QD-TTg dated on December 5, 2016 on approval the project of building and development health care network at the grassroots level in the new context (in Vietnamese). Quyết định phê duyệt đề án xây dựng và phát triển mạng lưới y tế cơ sở trong tình hình mới.

[ref16] Rocha KB , Rodriguez-Sanz M , Pasarin MI , Berra S , Gotsens M and Borrell C (2012) Assessment of primary care in health surveys: a population perspective. European Journal of Public Health 22, 14–19. 21470974 10.1093/eurpub/ckr014

[ref17] Safran DG , Kosinski M , Tarlov AR , Rogers WH , Taira DH , Lieberman N and Ware JE (1998) The Primary Care Assessment Survey: tests of data quality and measurement performance. Medical Care 36, 728–739. 9596063 10.1097/00005650-199805000-00012

[ref18] Seid M , Varni JW , Bermudez LO , Zivkovic M , Far MD , Nelson M and Kurtin PS (2001) Parents’ Perceptions of Primary Care: measuring parents’ experiences of pediatric primary care quality. Pediatrics 108, 264–270. 11483786 10.1542/peds.108.2.264

[ref19] Shi L , Macinko J , Starfield B , Politzer R , Wulu J and Xu J (2005a) Primary care, social inequalities and all-cause, heart disease and cancer mortality in US counties: a comparison between urban and non-urban areas. Public Health 119, 699–710. 15893346 10.1016/j.puhe.2004.12.007

[ref20] Shi L , Macinko J , Starfield B , Politzer R and Xu J (2005b) Primary care, race, and mortality in US states. Social Science and Medicine 61, 65–75. 15847962 10.1016/j.socscimed.2004.11.056

[ref21] Shi L , Starfield B and Xu J 2001 Validating the adult primary care assessment tool. Journal of Family Practice 50, 161.

[ref22] Starfield B (1991) Primary care and health. A cross-national comparison. JAMA 266, 2268–2271. 1920727

[ref23] Starfield B and Shi L (2002) Policy relevant determinants of health: an international perspective. Health Policy 60, 201–218. 11965331 10.1016/s0168-8510(01)00208-1

[ref24] Starfield B and Shi L (2009) Manual for the primary care assessment tools. Baltimore, MD: Johns Hopkins University.

[ref25] Starfield B , Shi L , Grover A and Macinko J (2005a) The effects of specialist supply on populations’ health: assessing the evidence. Health Affairs W5, 97–107. 10.1377/hlthaff.w5.97 15769797

[ref26] Starfield B , Shi L and Macinko J (2005b) Contribution of primary care to health systems and health. Milbank Quarterly 83, 457–502. 16202000 10.1111/j.1468-0009.2005.00409.x PMC2690145

[ref27] Veillard J , Cowling K , Bitton A , Ratcliffe H , Kimball M , Barkley S , Mercereau L , Wong E , Taylor C and Hirschhorn LR (2017) Better measurement for performance improvement in low-and middle-income countries: The Primary Health Care Performance Initiative (PHCPI) experience of conceptual framework development and indicator selection. The Milbank Quarterly 95, 836–883. 29226448 10.1111/1468-0009.12301 PMC5723717

[ref28] Vietnam Government (2016) Resolution No. 01/NQ-CP dated January 07, 2016 of the Government on major tasks and solutions for directing and administering the implementation of the 2016 socio-economic development plan and state budget estimates (in Vietnamese). Nghị quyết về những nhiệm vụ, giải pháp chủ yếu chỉ đạo điều hành thực hiện kế hoạch phát triển kinh tế - xã hội và dự toán ngân sách nhà nước năm 2016.

[ref29] Vietnam Ministry of Health (2013) Decision No.935/QĐ-BYT March 22, 2013, Approval of the project for the building and development of the family medicine clinic model in the period 2013–2020.

[ref30] Vietnam Prime Minister (2013) Decision No.92/QĐ-TTg January 9, 2013, Approval of the project for reducing overcrowding at hospitals in period 2013–2020, Hanoi, Vietnam.

[ref31] Wang W and Shi L (2014) Development and validation of the Tibetan primary care assessment tool. BioMed Research International 2014, 308739. 24967349 10.1155/2014/308739 PMC4055487

[ref32] Weidmer BA , Cleary PD , Keller S , Evensen C , Hurtado MP , Kosiak B , Gallagher PM , Levine R and Hays RD (2014) Development and evaluation of the CAHPS (Consumer Assessment of Healthcare Providers and Systems) survey for in-center hemodialysis patients. American Journal of Kidney Diseases 64, 753–760. 24998035 10.1053/j.ajkd.2014.04.021 PMC4356523

[ref33] WHO (2008) The world health report: Primary health care – Now more than ever.

[ref34] WHO and UNICEF (2018) Declaration of Astana, Global Conference on Primary Health Care, Astana, Kazakhstan, 25–26 October 2018.

[ref35] World Health Oganization (2016) *Sustainable Development Goals* Retrieved from https://www.un.org/sustainabledevelopment/health/ [accessed 30 April 2018].

[ref36] Yang H , Shi L , Lebrun LA , Zhou X , Liu J and Wang H (2013) Development of the Chinese primary care assessment tool: data quality and measurement properties. International Journal for Quality in Health Care 25, 92–105. 23175535 10.1093/intqhc/mzs072

[ref37] Zou Y , Zhang X , Hao Y , Shi L and Hu R (2015) General practitioners versus other physicians in the quality of primary care: a cross-sectional study in Guangdong Province, China. BMC Family Practice 16, 134. 26452648 10.1186/s12875-015-0349-z PMC4600296

