# Development and validation of the Vietnamese Primary Care Assessment Tool – provider version – ERRATUM

**DOI:** 10.1017/S1463423619000562

**Published:** 2019-08-15

**Authors:** Nguyen Thi Hoa, Anselme Derese, Jeffrey F. Markuns, Nguyen Minh Tam, Wim Peersman

**Keywords:** primary care quality, assessment, provider perspectives

Incorrect author details were given in the above mentioned article. Nguyen Minha Tam, ORCID number 0000-0003-3153-4606 should have been Nguyen Minh Tam, ORCID number 0000-0002-8505-677X

Cambridge University Press apologises for this error, which has since been rectified.
