# Supplementary material for: Development and validation of the Vietnamese Primary Care Assessment Tool – provider version
Source: Prim Health Care Res Dev. 2019 Jul 1;20:e86. doi: 10.1017/S1463423619000458 (PMC6609979; doi:10.1017/S1463423619000458)
Supplement: Supplementary file 1 [file S1463423619000458sup001.docx]

**DEVELOPMENT AND VALIDATION OF THE VIETNAMESE PRIMARY CARE ASSESSMENT TOOL - PROVIDER VERSION**

**Appendices**

**Table 1. Changes in the final translated questionnaires from the original PCAT provider**

| **Item code in the data set** | **Original question** | **Final translated question** |
| --- | --- | --- |
|  | **Type of practice (Check one.)**   1. Solo practice 2. Single specialty group practice 3. Multi-specialty group practice 4. Public health clinic 5. Community health clinic or neighborhood health center 6. Hospital clinic 7. Rural health clinic 8. Other (Please specify.) | **Where do you spend the majority of your time practicing? (Check one.)**   1. A commune health center 2. A ward health center 3. An outpatient department of a district hospital 4. An outpatient department of a provincial hospital 5. An outpatient department of a central hospital 6. A private clinic of a doctor 7. A private clinic of a group of doctors 8. Others (Please specify.) |
|  | **B. FIRST CONTACT** | |
| C5 | When your office is closed, do you have a phone number, patients can call when they get sick? | When your office is closed, can patients contact you or another doctor by phone when they get sick? |
| C8 | Can a patient easily get an appointment for routine check-ups at your office? | Can a patient easily get an appointment or make a visit for routine check-ups at your office? |
|  | **D. ONGOING CARE** | |
| D7 | Do you think you know the patients in your practice “very well”? | Do you think you know the patients in your practice “very well” (for example, both health condition and personal life)? |
|  | **E. COORDINATION** | |
| E1 | *Not available* | Does your office perform at least some laboratory tests? |
| E2 | Does your office phone or send patients the results of all lab tests? | Does your office share the results of the tests with patients (by phone call, mail, computer, or in person)? |
| E4 | When patients need a referral, do you discuss different places the family might go to get help with their problem? | When patients need to be referred to a specialist, do you discuss with them the options available to get help for their problem? |
| E8 | After the visit, do you talk with patients about the results of visits with the specialist or special service? | Do you talk with your patients about their visit to specialists or special service and the results of those visits? |
|  | **F. COORDINATION (information system)** | |
| F1 | *Not available* | Do all patients have a medical record at the facility? |
| F2 | Are patients expected to bring their medical records, such as immunizations or medical care they received in the past? | Do patients have a medical record or booklet (such as an y ba) that they keep with them and bring to visits? |
| F4 | Are patient records available when you see patients? (either personal or facility records) | Are patient records available when you see patients? |
| F9 | Medication lists in patients’ records | List of medications patients are taking |
|  | **G. COMPREHENSIVENESS (SERVICES AVAILABLE)** | |
| G1 | Nutrition counselling by a nutrition specialist | Nutrition counselling |
| G3 | Eligibility screening for social service programs or benefits | Assistance with obtaining available social service programs/benefits |
| G4 | Dental check-ups | Dental check-ups and Dental treatments |
|  | Dental treatments |  |
| G6 | Tests for lead poisoning | Substance or drug abuse counselling or treatment |
| G14 | Splinting for a sprained ankle | Temporarily fix a broken bone |
| G15 | Wart removal | Gastric catheter insertion |
| G16 | Pap smears | Pap smears, cervical cancer screening |
| G17 | Rectal exam or sigmoidoscopy | Rectal exam or colon cancer screening |
| G20 | Removal of an ingrown toenail | Shoulder reduction |
| G21 | Advice on advance directives | Advice on End of life issues/Palliative Care |
| G23 | Suggestions on nursing home care | Postpartum care of umbilical cord |
| G24 | WIC services (supplemental milk and food program) | Monitoring of normal pregnancy |
|  | **H. COMPREHENSIVENESS (SERVICES PROVIDED)** | |
| H3 | Seat belt use | Seat belt or helmet use |
| H15 | **Safety issues for children under 6**: teaching them to cross the street safely, and using child safety seats in cars | Safety issues for children under 6: (injury prevention, fire and electricity safety, food safety, drowning prevention) |
| H16 | **Safety issues for children between 6 and 12:** staying away from guns, and using seatbelts and bicycle helmets | Safety issues for children between 6 and 12: (including using helmets and/or seatbelts) |
|  | **I. FAMILY-CENTEREDNESS** | |
| I7 | Discussion of living conditions, e.g., working refrigerator, heat | Discussion of living conditions, (for example: clean water, latrine/toilet, stress at work or home) |
|  | **J. COMMUNITY ORIENTATION** | |
| J17 | Community health workers | Community/village health workers |
| J18 | Have a consumer on the board of directors or advisory committee | Gather feedback from patients about health staff performance |
| J21 | Linkages with religious organizations/ services | Linkages with religious organizations |
| J23 | Outreach workers | Village health workers |
|  | **K. CULTURALLY COMPETENT** | |
| K1 | Can your office communicate with people who do not speak English well? | Can someone in your office communicate well with patients who speak another language (such as patients from ethnic minority groups)? |
| K2 | If needed, do you take into account a family’s special beliefs about health care or use of folk medicine, such as herbs/homemade medicines? | Do you take into account a family’s special beliefs about health care or use of folk medicine, such as herbs/homemade medicines? |
| K3 | If needed, do you take into account a family’s request to use alternative treatment, such as homeopathy or acupuncture? | Do you take into account a family’s request to use alternative treatment, such as homeopathy or acupuncture? |

**Table 2. Item correlation with domain scores after review (item convergent validity and item discriminant validity)**

| **Item code in the data set** | **Domain** | **First Contact** | **Ongoing Care** | **Coordination** | **Coordination (information system)** | **Comprehensiveness (Services available)** | **Comprehensiveness (Services provided)** | **FamilyCenteredness** | **Community Orientation** | **Culturally Competent** |
| --- | --- | --- | --- | --- | --- | --- | --- | --- | --- | --- |
|  | **B. FIRST CONTACT (9 items)** |  |  |  |  |  |  |  |  |  |
| C1 | Is your office open on Saturday or Sunday? | **0.75** | 0.08 | 0.19 | 0.21 | 0.26 | 0.16 | 0.10 | 0.14 | 0.16 |
| C2 | Is your office open on at least some weekday evenings until 8 PM? | **0.73** | 0.00 | 0.10 | 0.09 | 0.09 | -0.04 | -0.04 | 0.04 | 0.14 |
| C3 | When your office is open, and patients get sick, would someone from your office see them that day? | **0.45** | 0.20 | 0.17 | 0.27 | 0.25 | 0.16 | 0.07 | 0.11 | 0.19 |
| C4 | When the office is open, can patients get advice quickly over the phone when they think they need it? | **0.62** | 0.27 | 0.21 | 0.19 | 0.30 | 0.21 | 0.16 | 0.19 | 0.20 |
| C5 | When your office is closed, can patients contact you or another doctor by phone when they get sick? | **0.70** | 0.25 | 0.16 | 0.14 | 0.29 | 0.21 | 0.20 | 0.21 | 0.21 |
| C6 | If your office is closed on Saturday or Sunday and patients get sick, would someone from your office be able to see them that day? | **0.75** | 0.15 | 0.23 | 0.18 | 0.24 | 0.13 | 0.13 | 0.16 | 0.21 |
| C7 | When your office is closed during the night and patients get sick, would someone from your office be able to see them that night? | **0.66** | 0.18 | 0.17 | 0.20 | 0.17 | 0.07 | 0.11 | 0.17 | 0.23 |
| C8 | Can a patient easily get an appointment or make a visit for routine check-ups at your office? | **0.56** | 0.34 | 0.30 | 0.20 | 0.35 | 0.26 | 0.25 | 0.27 | 0.21 |
| C9 | On average, do patients have to wait more than 30 minutes after arriving before they are examined by the doctor or nurse? * | Not assessed | | | | | | | | |
|  | **D. ONGOING CARE (13 items)** |  | | | | | | | | |
| D1 | At your office, do patients see the same clinician each time they make a visit? * | Not assessed | | | | | | | | |
| D2 | Can you understand the questions that your patients ask you? | 0.16 | **0.49** | 0.15 | 0.20 | 0.23 | 0.16 | 0.20 | 0.19 | 0.25 |
| D3 | Do you think your patients understand what you ask them or say to them? | 0.08 | **0.57** | 0.14 | 0.21 | 0.30 | 0.27 | 0.20 | 0.22 | 0.16 |
| D4 | If patients have a question, can they call and talk to the doctor or nurse who knows them best? | 0.36 | **0.52** | 0.31 | 0.19 | 0.23 | 0.18 | 0.29 | 0.18 | 0.27 |
| D5 | Do you think you give patients enough time to talk about their worries or problems? | 0.29 | **0.54** | 0.35 | 0.29 | 0.33 | 0.31 | 0.34 | 0.30 | 0.21 |
| D6 | Do you think your patients feel comfortable telling you about their worries or problems? | 0.17 | **0.53** | 0.30 | 0.27 | 0.34 | 0.39 | 0.44 | 0.22 | 0.21 |
| D7 | Do you think you know the patients in your practice “very well” (for example, both health condition and personal life)? | 0.02 | **0.59** | 0.36 | 0.16 | 0.31 | 0.31 | 0.41 | 0.26 | 0.24 |
| D8 | Do you know who lives with each of your patients? | 0.15 | **0.61** | 0.32 | 0.04 | 0.31 | 0.29 | 0.33 | 0.31 | 0.19 |
| D9 | Do you think you understand what problems are most important to the patients you see? | 0.09 | **0.66** | 0.36 | 0.25 | 0.47 | 0.38 | 0.38 | 0.39 | 0.37 |
| D10 | Do you think you know each patient’s complete medical history? | 0.05 | **0.70** | 0.35 | 0.31 | 0.29 | 0.38 | 0.30 | 0.34 | 0.24 |
| D11 | Do you think you know each patient’s work or employment? | 0.07 | **0.69** | 0.39 | 0.29 | 0.40 | 0.43 | 0.33 | 0.47 | 0.30 |
| D12 | Would you know if patients had trouble getting or paying for a prescribed medication? | 0.22 | **0.66** | 0.48 | 0.31 | 0.34 | 0.30 | 0.33 | 0.31 | 0.20 |
| D13 | Do you know all the medications that your patients are taking? | 0.16 | **0.61** | 0.37 | 0.29 | 0.37 | 0.36 | 0.33 | 0.33 | 0.16 |
|  | **E. COORDINATION (7 items)** |  |  |  |  |  |  |  |  |  |
| E2 | Does your office share the results of the tests with patients (by phone call, mail, computer, or in person)? | 0.16 | 0.27 | **0.45** | 0.14 | 0.33 | 0.11 | 0.08 | 0.06 | 0.02 |
| E3 | Do you think you know about all the visits that your patients make to specialists or special services? | 0.26 | 0.41 | **0.61** | 0.45 | 0.40 | 0.44 | 0.43 | 0.38 | 0.38 |
| E4 | When patients need to be referred to a specialist, do you discuss with them the options available to get help for their problem? | 0.22 | 0.52 | **0.69** | 0.37 | 0.48 | 0.47 | 0.43 | 0.36 | 0.29 |
| E5 | Does someone at your office help the patient make the appointment for the referral visit? | 0.11 | 0.21 | **0.54** | 0.19 | 0.30 | 0.37 | 0.37 | 0.31 | 0.30 |
| E6 | When patients are referred, do you give them any written information to take to the specialist? | 0.26 | 0.32 | **0.75** | 0.35 | 0.38 | 0.33 | 0.33 | 0.34 | 0.20 |
| E7 | Do you receive useful information about your referred patients back from the specialists or special services? | 0.12 | 0.21 | **0.63** | 0.20 | 0.14 | 0.18 | 0.30 | 0.21 | 0.24 |
| E8 | Do you talk with your patients about their visit to specialists and the results of the visits to the specialist or special service? | 0.10 | 0.43 | **0.66** | 0.31 | 0.26 | 0.33 | 0.36 | 0.29 | 0.30 |
|  | **F. COORDINATION (information system) (9 items)** |  |  |  |  |  |  |  |  |  |
| F1 | Do all patients have a medical record at the facility? | 0.11 | 0.16 | 0.08 | **0.60** | 0.07 | 0.12 | 0.10 | 0.14 | 0.06 |
| F2 | Do patients have a medical record or booklet (such as an y ba) that they keep with them and bring to visits? | 0.20 | 0.18 | 0.26 | **0.47** | 0.15 | 0.16 | 0.07 | 0.16 | 0.09 |
| F3 | Would you allow patients to look at their medical records at your office if they wanted to? | 0.07 | 0.20 | 0.32 | **0.35** | 0.16 | 0.13 | 0.14 | 0.10 | 0.03 |
| F4 | Are patient records available when you see patients? (either personal or facility records) | 0.26 | 0.20 | 0.29 | **0.67** | 0.16 | 0.23 | 0.14 | 0.27 | 0.10 |
|  | **Do you use the following methods to assure that indicated services are provided**? |  |  |  |  |  |  |  |  |  |
| F5 | Flow sheets in patients’ charts for lab results | 0.10 | 0.26 | 0.40 | **0.65** | 0.29 | 0.37 | 0.37 | 0.30 | 0.29 |
| F6 | Printed guidelines in patients’ records | 0.18 | 0.24 | 0.29 | **0.78** | 0.33 | 0.39 | 0.28 | 0.37 | 0.22 |
| F7 | Periodic review of patient medical records | 0.25 | 0.34 | 0.40 | **0.82** | 0.43 | 0.45 | 0.42 | 0.46 | 0.33 |
| F8 | Problem lists in patients’ records | 0.15 | 0.36 | 0.39 | **0.82** | 0.39 | 0.44 | 0.35 | 0.43 | 0.28 |
| F9 | List of medications patients are taking | 0.26 | 0.33 | 0.41 | **0.78** | 0.38 | 0.34 | 0.29 | 0.27 | 0.20 |
|  | **G. COMPREHENSIVENESS (SERVICES AVAILABLE) 24 items** |  |  |  |  |  |  |  |  |  |
|  | **If patients need any of the following services, would they be able to get them on-site at your office?** |  |  |  |  |  |  |  |  |  |
| G1 | Nutrition counselling | 0.37 | 0.42 | 0.37 | 0.35 | **0.57** | 0.44 | 0.32 | 0.34 | 0.32 |
| G2 | Immunizations | 0.34 | 0.42 | 0.28 | 0.32 | **0.56** | 0.28 | 0.17 | 0.33 | 0.23 |
| G3 | Assistance with obtaining available social service programs/benefits | 0.23 | 0.37 | 0.34 | 0.21 | **0.49** | 0.37 | 0.37 | 0.32 | 0.28 |
| G4 | Dental check-ups and Dental treatments | 0.15 | 0.30 | 0.36 | 0.22 | **0.52** | 0.40 | 0.33 | 0.26 | 0.25 |
| G5 | Family planning or birth control services | 0.25 | 0.48 | 0.26 | 0.28 | **0.64** | 0.43 | 0.29 | 0.43 | 0.27 |
| G6 | Substance or drug abuse counselling or treatment | 0.24 | 0.46 | 0.34 | 0.33 | **0.68** | 0.50 | 0.29 | 0.38 | 0.20 |
| G7 | Counselling for behaviour or mental health problems | 0.32 | 0.40 | 0.37 | 0.29 | **0.72** | 0.51 | 0.37 | 0.43 | 0.32 |
| G8 | Counselling and treating alcoholism | 0.15 | 0.31 | 0.37 | 0.33 | **0.68** | 0.51 | 0.38 | 0.38 | 0.35 |
| G9 | Suturing for a minor laceration | 0.34 | 0.40 | 0.35 | 0.18 | **0.64** | 0.32 | 0.25 | 0.32 | 0.19 |
| G10 | Counselling and testing for HIV/AIDS | 0.23 | 0.31 | 0.31 | 0.27 | **0.63** | 0.40 | 0.26 | 0.34 | 0.22 |
| G11 | Remove fluid from behind the eardrum to check for infection | 0.18 | 0.23 | 0.25 | 0.09 | **0.59** | 0.29 | 0.33 | 0.40 | 0.31 |
| G12 | Vision screening | 0.24 | 0.29 | 0.35 | 0.24 | **0.68** | 0.46 | 0.35 | 0.31 | 0.29 |
| G13 | Allergy shots | 0.18 | 0.46 | 0.31 | 0.24 | **0.68** | 0.48 | 0.37 | 0.36 | 0.21 |
| G14 | Temporarily fix a broken bone | 0.28 | 0.36 | 0.36 | 0.30 | **0.72** | 0.42 | 0.35 | 0.43 | 0.29 |
| G15 | Gastric catheter insertion | 0.15 | 0.13 | 0.27 | 0.22 | **0.49** | 0.33 | 0.39 | 0.34 | 0.34 |
| G16 | Pap smears, cervical cancer screening | 0.06 | 0.08 | 0.20 | 0.19 | **0.35** | 0.19 | 0.15 | 0.25 | 0.29 |
| G17 | Colon cancer screening | 0.04 | 0.14 | 0.32 | 0.26 | **0.37** | 0.27 | 0.30 | 0.32 | 0.34 |
| G18 | Smoking counselling | 0.12 | 0.42 | 0.33 | 0.24 | **0.70** | 0.55 | 0.43 | 0.46 | 0.38 |
| G19 | Prenatal care | 0.31 | 0.42 | 0.42 | 0.22 | **0.73** | 0.46 | 0.35 | 0.32 | 0.32 |
| G20 | Shoulder reduction | 0.11 | 0.18 | 0.32 | 0.15 | **0.65** | 0.38 | 0.29 | 0.28 | 0.28 |
| G21 | Advice on End of life issues/Palliative Care | 0.06 | 0.21 | 0.28 | 0.13 | **0.56** | 0.39 | 0.42 | 0.35 | 0.30 |
| G22 | Advice on preparing for changes consequent to aging | 0.18 | 0.35 | 0.37 | 0.38 | **0.62** | 0.57 | 0.51 | 0.40 | 0.35 |
| G23 | Postpartum care of umbilical cord | 0.29 | 0.39 | 0.32 | 0.25 | **0.70** | 0.46 | 0.41 | 0.32 | 0.31 |
| G24 | Monitoring of normal pregnancy | 0.35 | 0.35 | 0.29 | 0.28 | **0.64** | 0.36 | 0.25 | 0.35 | 0.21 |
|  | **H. COMPREHENSIVENESS (SERVICES PROVIDED) 17 items** |  |  |  |  |  |  |  |  |  |
|  | **If your office serves all ages, please answer all questions in this section (H1 – H17). If your office serves only children, do not answer questions H3 – H12. If your office serves only adults, do not answer questions H13 – H17.** |  |  |  |  |  |  |  |  |  |
|  | **Are the following subjects discussed with patients?** |  |  |  |  |  |  |  |  |  |
| H1 | Nutritional/non-nutritional foods or getting enough sleep | 0.19 | 0.45 | 0.36 | 0.31 | 0.48 | **0.64** | 0.51 | 0.48 | 0.32 |
| H2 | Home safety, such as storing medicines safely | 0.18 | 0.35 | 0.26 | 0.34 | 0.34 | **0.51** | 0.35 | 0.37 | 0.19 |
|  | **Questions H3 – H12 apply to adults only (ages 18 and older).** |  |  |  |  |  |  |  |  |  |
|  | **Are the following subjects discussed with patients?** |  |  |  |  |  |  |  |  |  |
| H3 | Seat belt or helmet use | 0.12 | 0.30 | 0.20 | 0.22 | 0.33 | **0.63** | 0.48 | 0.39 | 0.42 |
| H4 | Handling family conflicts | 0.09 | 0.32 | 0.38 | 0.25 | 0.33 | **0.55** | 0.51 | 0.41 | 0.38 |
| H5 | Advice about appropriate exercise | 0.11 | 0.24 | 0.29 | 0.21 | 0.37 | **0.63** | 0.50 | 0.36 | 0.34 |
| H6 | Cholesterol levels | 0.11 | 0.21 | 0.39 | 0.29 | 0.36 | **0.54** | 0.33 | 0.23 | 0.20 |
| H7 | Medications being taken | 0.13 | 0.34 | 0.31 | 0.19 | 0.44 | **0.61** | 0.49 | 0.36 | 0.33 |
| H8 | Exposure to harmful substances at home, work, or in their neighbourhood | 0.14 | 0.49 | 0.34 | 0.32 | 0.58 | **0.74** | 0.54 | 0.54 | 0.38 |
|  | Gun availability, storage, safety* | Not assessed | | | | | | | | |
| H9 | Prevention of hot water burns | 0.16 | 0.43 | 0.38 | 0.30 | 0.61 | **0.81** | 0.61 | 0.59 | 0.49 |
| H10 | Prevention of falls | 0.07 | 0.40 | 0.36 | 0.31 | 0.63 | **0.80** | 0.60 | 0.56 | 0.47 |
| H11 | Prevention of osteoporosis or fragile bones in females | 0.09 | 0.26 | 0.42 | 0.37 | 0.52 | **0.70** | 0.51 | 0.46 | 0.35 |
| H12 | Care for common menstrual or menopausal problems | 0.13 | 0.30 | 0.46 | 0.41 | 0.55 | **0.73** | 0.53 | 0.41 | 0.40 |
|  | **Questions H13– H17 apply to children only (under age 18)**  **Are the following subjects discussed with the child and parent/guardian?** |  |  |  |  |  |  |  |  |  |
| H13 | Ways to handle problems with child’s behaviour | 0.22 | 0.35 | 0.44 | 0.37 | 0.52 | **0.77** | 0.64 | 0.55 | 0.42 |
| H14 | Changes in growth and behaviour that parents can expect at certain ages | 0.17 | 0.35 | 0.39 | 0.34 | 0.42 | **0.71** | 0.63 | 0.56 | 0.42 |
| H15 | Safety issues for children under 6: (injury prevention, fire and electricity safety, food safety, drowning prevention) | 0.18 | 0.42 | 0.37 | 0.22 | 0.48 | **0.76** | 0.66 | 0.59 | 0.44 |
| H16 | Safety issues for children between 6 and 12: (including using helmets and/or seatbelts) | 0.11 | 0.35 | 0.32 | 0.27 | 0.39 | **0.74** | 0.66 | 0.46 | 0.44 |
| H17 | Safety issues for children over 12: safe sex, saying no to drugs, not drinking and driving | 0.10 | 0.39 | 0.32 | 0.38 | 0.39 | **0.70** | 0.66 | 0.40 | 0.38 |
|  | **I. FAMILY-CENTEREDNESS 13 items** |  |  |  |  |  |  |  |  |  |
| I1 | Does your office ask patients about their ideas and opinions when planning treatment and care for the patient or family member? | 0.17 | 0.36 | 0.41 | 0.24 | 0.24 | 0.43 | **0.64** | 0.41 | 0.39 |
| I2 | Does your office ask about illnesses or problems that might run in the patients’ families? | 0.15 | 0.43 | 0.38 | 0.33 | 0.47 | 0.59 | **0.71** | 0.48 | 0.38 |
| I3 | Is your office willing and able to meet with family members to discuss a health or family problem? | 0.28 | 0.47 | 0.40 | 0.15 | 0.36 | 0.46 | **0.61** | 0.43 | 0.33 |
|  | **Are the following included as a routine part of your health assessment?** |  |  |  |  |  |  |  |  |  |
|  | Use of familiograms, family APGAR* | Not assessed | | | | | | | | |
| I4 | Discussion of family health risk factors, e.g., genetics | 0.13 | 0.46 | 0.39 | 0.28 | 0.36 | 0.54 | **0.67** | 0.56 | 0.38 |
| I5 | Discussion of family economic resources | 0.09 | 0.40 | 0.38 | 0.23 | 0.37 | 0.63 | **0.77** | 0.50 | 0.46 |
| I6 | Discussion of social risk factors, e.g., loss of employment | 0.05 | 0.32 | 0.44 | 0.27 | 0.31 | 0.55 | **0.75** | 0.44 | 0.52 |
| I7 | Discussion of living conditions, (for example: clean water, latrine/toilet, stress at work or home) | 0.16 | 0.42 | 0.33 | 0.27 | 0.51 | 0.63 | **0.76** | 0.59 | 0.43 |
| I8 | Discussion of health status of other family members | 0.14 | 0.39 | 0.38 | 0.26 | 0.43 | 0.60 | **0.77** | 0.58 | 0.39 |
| I9 | Discussion of parenting | 0.05 | 0.37 | 0.38 | 0.32 | 0.45 | 0.68 | **0.78** | 0.52 | 0.48 |
| I10 | Assessment of signs of child abuse | 0.01 | 0.30 | 0.36 | 0.25 | 0.38 | 0.58 | **0.77** | 0.54 | 0.46 |
| I11 | Assessment of indications of family in crisis | 0.02 | 0.27 | 0.35 | 0.24 | 0.39 | 0.57 | **0.75** | 0.46 | 0.42 |
| I12 | Assessment of impact of patient’s health on family functioning | 0.10 | 0.38 | 0.42 | 0.35 | 0.41 | 0.67 | **0.78** | 0.56 | 0.41 |
| I13 | Assessment of development level | 0.29 | 0.38 | 0.40 | 0.37 | 0.50 | 0.54 | **0.63** | 0.55 | 0.51 |
|  | **J. COMMUNITY ORIENTATION (21 items)** |  |  |  |  |  |  |  |  |  |
| J1 | Does your office make home visits? * | 0.05 | 0.30 | 0.23 | 0.00 | 0.30 | 0.40 | 0.48 | **0.44** | 0.28 |
| J2 | Do you think your office has adequate knowledge about the health problems of the communities you serve? * | 0.18 | 0.24 | 0.31 | 0.14 | 0.20 | 0.30 | 0.48 | **0.41** | 0.36 |
| J3 | Does your office get opinions and ideas from people that might help to provide better health care? | 0.19 | 0.29 | 0.26 | 0.28 | 0.33 | 0.36 | 0.43 | **0.59** | 0.45 |
| J4 | Is your office able to change health care services or programs in response to specific health problems in the communities? * | 0.10 | 0.16 | 0.26 | 0.24 | 0.32 | 0.40 | 0.45 | 0.45 | 0.23 |
|  | **Does your office use the following types of data to determine what programs/services are needed by the communities you serve?** |  |  |  |  |  |  |  |  |  |
| J5 | Mortality data (data on deaths) | 0.12 | 0.39 | 0.21 | 0.22 | 0.38 | 0.42 | 0.39 | **0.47** | 0.27 |
| J6 | Public health communicable disease data (e.g., STDs, TB) | 0.20 | 0.43 | 0.22 | 0.25 | 0.39 | 0.42 | 0.35 | **0.53** | 0.33 |
| J7 | Community immunization rates | 0.25 | 0.44 | 0.27 | 0.35 | 0.43 | 0.34 | 0.37 | **0.51** | 0.29 |
| J8 | Public health data on health or occupational hazards | 0.08 | 0.37 | 0.26 | 0.26 | 0.48 | 0.42 | 0.42 | **0.59** | 0.31 |
| J9 | Clinical data from your practice | 0.16 | 0.29 | 0.28 | 0.36 | 0.32 | 0.42 | 0.39 | **0.60** | 0.34 |
|  | **Does your office use the following methods to monitor and/or evaluate the effectiveness of services/programs?** |  |  |  |  |  |  |  |  |  |
| J11 | Surveys of your patients | 0.14 | 0.26 | 0.30 | 0.31 | 0.33 | 0.39 | 0.41 | **0.73** | 0.32 |
| J12 | Community surveys | 0.06 | 0.23 | 0.28 | 0.23 | 0.32 | 0.39 | 0.37 | **0.74** | 0.34 |
| J13 | Feedback from community organizations or community advisory boards | 0.08 | 0.35 | 0.33 | 0.31 | 0.37 | 0.46 | 0.52 | **0.79** | 0.43 |
| J14 | Feedback from your practice staff | 0.20 | 0.34 | 0.34 | 0.34 | 0.37 | 0.47 | 0.50 | **0.73** | 0.38 |
| J15 | Analysis of local data or vital statistics | 0.05 | 0.34 | 0.33 | 0.30 | 0.29 | 0.46 | 0.51 | **0.76** | 0.42 |
| J16 | Systematic evaluations of your programs and services provided | 0.13 | 0.39 | 0.44 | 0.34 | 0.42 | 0.53 | 0.56 | **0.77** | 0.42 |
| J17 | Community/village health workers | 0.23 | 0.31 | 0.16 | 0.22 | 0.28 | 0.34 | 0.40 | **0.63** | 0.33 |
| J18 | Gather feedback from patients about health staff performance | 0.07 | 0.30 | 0.26 | 0.24 | 0.28 | 0.40 | 0.43 | **0.67** | 0.36 |
|  | **Does your office use any of the following activities to reach out to populations in the communities you serve?** |  |  |  |  |  |  |  |  |  |
| J20 | Networking with state and local agencies involved with culturally diverse groups | 0.18 | 0.20 | 0.22 | 0.27 | 0.33 | 0.39 | 0.47 | **0.64** | 0.46 |
| J21 | Linkages with religious organizations | 0.12 | 0.24 | 0.34 | 0.24 | 0.34 | 0.38 | 0.41 | **0.67** | 0.37 |
| J22 | Involvement with neighbourhood groups/ community leaders | 0.24 | 0.23 | 0.29 | 0.35 | 0.45 | 0.49 | 0.54 | **0.67** | 0.46 |
| J23 | Village health workers* | 0.29 | 0.29 | 0.15 | 0.19 | 0.42 | 0.29 | 0.33 | **0.38** | 0.27 |
|  | **K. CULTURALLY COMPETENT (9 items)** |  |  |  |  |  |  |  |  |  |
| K1 | Can someone in your office communicate well with patients who speak another language (such as patients from ethnic minority groups)? | 0.19 | 0.34 | 0.07 | 0.12 | 0.19 | 0.21 | 0.24 | 0.25 | **0.58** |
| K2 | Do you take into account a family’s special beliefs about health care or use of folk medicine, such as herbs/homemade medicines? | 0.28 | 0.25 | 0.29 | 0.22 | 0.39 | 0.35 | 0.35 | 0.34 | **0.55** |
| K3 | Do you take into account a family’s request to use alternative treatment, such as homeopathy or acupuncture? | 0.33 | 0.42 | 0.45 | 0.23 | 0.46 | 0.43 | 0.47 | 0.38 | **0.49** |
|  | **Does your office use any of the following methods to address the cultural diversity in your patient population?** |  |  |  |  |  |  |  |  |  |
| K4 | Training of staff by outside instructors | 0.15 | 0.19 | 0.35 | 0.17 | 0.26 | 0.37 | 0.43 | 0.48 | **0.68** |
| K5 | In-service programs presented by staff | 0.11 | 0.08 | 0.29 | 0.23 | 0.31 | 0.38 | 0.39 | 0.41 | **0.64** |
| K6 | Use of culturally-sensitive (language, visual images, religious customs) materials/pamphlets | 0.13 | 0.22 | 0.26 | 0.17 | 0.30 | 0.42 | 0.46 | 0.48 | **0.73** |
| K7 | Staff reflecting the cultural diversity of the population served | 0.24 | 0.30 | 0.22 | 0.20 | 0.28 | 0.43 | 0.42 | 0.41 | **0.73** |
| K8 | Translators/interpreters | 0.10 | 0.18 | 0.11 | 0.08 | 0.16 | 0.25 | 0.29 | 0.21 | **0.65** |
| K9 | Planning of services that reflect cultural diversity | 0.18 | 0.20 | 0.21 | 0.18 | 0.30 | 0.41 | 0.45 | 0.45 | **0.73** |
| **: removed from the final questionnaires* | | | | | | | | | | |
